# Cardio4Health Study, a Cardiac Telerehabilitation Pilot Program Aimed at Patients After an Ischemic Event: Cross-sectional Study

**DOI:** 10.2196/44179

**Published:** 2023-04-24

**Authors:** Margarita Calvo-López, Raquel Arranz Tolós, Josefa Marin Expósito, Domenico Gruosso, Rut Andrea, Mercè Roque, Carles Falces, Gemma Yago, Judith Saura Araguas, Nuria Pastor, Marta Sitges, Maria Sanz-de la Garza

**Affiliations:** 1 Cardiovascular Clinical Institute Hospital Clínic Barcelona Spain; 2 Cardiovascular Institute August Pi I Sunyer Biomedical Research Institute (IDIBAPS) Barcelona Spain; 3 HumanITcare Small Medium Enterprise (SME) Barcelona Spain

**Keywords:** cardiac rehabilitation, web-based platform, telemedicine, remote care, ischemic heart disease

## Abstract

**Background:**

Center-based cardiac rehabilitation programs (CRPs) reduce morbidity and mortality after an ischemic cardiac event; however, they are widely underused. Home-based CRP has emerged as an alternative to improve patient adherence; however, its safety and efficacy remain unclear, especially for older patients and female patients.

**Objective:**

This study aimed to develop a holistic home-based CRP for patients with ischemic heart disease and evaluate its safety and impact on functional capacity, adherence to a healthy lifestyle, and quality of life.

**Methods:**

The 8-week home-based CRP included patients of both sexes, with no age limit, who had overcome an acute myocardial infarction in the previous 3 months, had a left ventricular ejection fraction of ≥40%, and had access to a tablet or mobile device. The CRP was developed using a dedicated platform designed explicitly for this purpose and included 3 weekly exercise sessions combining tailored aerobic and strength training and 2 weekly educational session focused on lifestyle habits, therapeutic adherence, and patient empowerment.

**Results:**

We initially included 62 patients, of whom 1 was excluded for presenting with ventricular arrhythmias during the initial stress test, 5 were excluded because of incompatibility, and 6 dropped out because of a technological barrier. Ultimately, 50 patients completed the program: 85% (42/50) were male, with a mean age of 58.9 (SD 10.3) years, a mean left ventricular ejection fraction of 52.1% (SD 6.72%), and 25 (50%) New York Heart Association functional class I and 25 (50%) New York Heart Association II-III. The CRP significantly improved functional capacity (+1.6 metabolic equivalent tasks), muscle strength (arm curl test +15.5% and sit-to-stand test +19.7%), weekly training volume (+803 metabolic equivalent tasks), adherence to the Mediterranean diet, emotional state (anxiety), and quality of life. No major complications occurred, and adherence was excellent (>80%) in both the exercise and educational sessions. In the subgroup analysis, CRP showed equivalent beneficial effects irrespective of sex and age. In addition, patient preferences for CRP approaches were equally distributed, with one-third (14/50, 29%) of the patients preferring a face-to-face CRP, one-third (17/50, 34%) preferring a telematic CRP, and one-third (18/50, 37%) preferring a hybrid approach. Regarding CRP duration, 63% (31/50) of the patients considered it adequate, whereas the remaining 37% (19/50) preferred a longer program.

**Conclusions:**

A holistic telematic CRP dedicated to patients after an ischemic cardiac event, irrespective of sex and age, is safe and, in our population, has achieved positive results in improving maximal aerobic capacity, weekly training volume, muscle strength, quality of life, compliance with diet, and anxiety symptoms. The preference for a center- or home-based CRP approach is diverse among the study population, emphasizing the need for a tailored CRP to improve adherence and completion rates.

## Introduction

### Background

The cardiac rehabilitation program (CRP) is a comprehensive, multidisciplinary, secondary prevention program for optimizing cardiovascular health. Multifaceted programs can address the psychosocial, physical, nutritional, and emotional aspects of cardiovascular health. Supervised CRP is beneficial and safe for different populations of patients with cardiovascular diseases, such as those who have experienced an ischemic event [1,2], cardiac surgery [3], or heart failure [4]. Thus, international guidelines recommend, with a maximum level of evidence *1A*, CRPs for cardiovascular pathologies [5,6].

The beneficial effects of CRP on the abovementioned cardiovascular diseases include a considerable improvement of functional capacity [7], greater adherence to a healthy lifestyle [8], and lower incidence of disease-associated anxiety and depression [9], all of which improve the quality of life of the patient [10]. Furthermore, CRP reduces cardiovascular morbidity and mortality mid- to long-term in patients who have experienced an ischemic event [1,11]. Over the last decade, supervised CRP has been increasingly used for patients who have experienced an ischemic event; however, the percentage of patients benefiting from these programs is still far from 100% [12].

CRP implementation has been mainly hindered by the associated costs [12] and low adherence of eligible patients [13], which may be because of their incompatible working hours [14]. Many patients who have experienced an ischemic event continue working and cannot attend the CRP, which is usually performed during the usual consultation hours (morning and half an afternoon). Other variables associated with low adherence are difficulty traveling to the CRP center, low socioeconomic status [13], and frailty with concomitant pluripathology. In contrast, female individuals traditionally have a higher familial burden and lower physical activity levels than male individuals, leading to a decline in their reduced CRP participation [15].

Technological advancement over the last decade has allowed the development of a novel web-based CRP designated as cardiac telerehabilitation. Such programs are effective and safe for patients following an ischemic event [16]. However, most of the currently available evidence comes from selected populations, including middle-aged male patients with a high degree of motivation to undertake the program and excluding those expected to have lower adherence, such as female patients [15], patients aged >75 years, rural residents [17], and patients with multiple comorbidities [18].

The development of telematic CRP is significant in the past scenario of the COVID-19 pandemic. Such programs would allow the continuation of CRPs while minimizing the contagion risk.

However, many CRPs focus only on physical training (PT), disregarding interventions for the emotional sphere [19], nutrition [20], and medication adherence [8], which are essential for improving patient adherence to a healthy lifestyle. In addition, PT in several CRPs includes only aerobic exercise, despite the safety and synergistic effect of strength training in improving functional capacity [21].

In this study, we developed a web-based cardiac telerehabilitation platform, specifically designed for patients following ischemic events. The holistic telematic CRP includes PT and interventions to improve the emotional sphere and adherence to a healthy lifestyle.

### Objectives

*The primary objective* of this pilot study was to evaluate the result of the cardiac telerehabilitation program in terms of increasing the aerobic functional capacity, evaluated using the metabolic equivalent tasks (METs) achieved in a maximal exercise test, and strength, measured through repetitions of sit-to-stand and arm curl tests.

*The secondary objectives* were to assess the following:

- Feasibility of the program and the designed platform
- Safety of the program: assessing whether major or minor events (cardiovascular or other types, if relevant) occurred within 24 hours after PT
- Quality of life using the European health questionnaire combining quantity and quality of life (EuroQoL) [22]
- Adherence to the program
- Adherence to the Mediterranean diet using the Mediterranean diet and lifestyle (*Prevención con Dieta Mediterránea* [PREDIMED]) questionnaire [23]
- Emotional state using the Hospital Anxiety and Depression Scale (HADS) [24].

## Methods

### Study Design

This was a single-center prospective study with participant recruitment from the Cardiovascular Institute of the Hospital Clínic (*Institut d’Investigacions Biomèdiques August Pi i Sunyer*) at the University of Barcelona, Spain, conducted between December 2020 and January 2022.

All participants provided verbal and written consent.

The variables listed below were included on the web platform used for the cardiac telerehabilitation program. The program researchers collected data and were anonymous in all cases.

### Ethics Approval

The Ethical Committe for Research on Medicines at the Hospital Clinic of the Universitat de Barcelona approved this study in November 2020 (ethics ID: HCB/2020/1021), which aligned with the Helsinki Declaration.

### Variables Analyzed

#### Variables Related to the Study Population

The following data were collected from the study population:

- Age
- Sex
- Weight, height, BMI, and abdominal circumference
- Family history
- History of cardiovascular pathology and comorbidities
- Socioeconomic data: employment status and educational level
- Systolic and diastolic blood pressures were measured under normal daily conditions (nonfasting state and with antihypertensive treatment administered if previously prescribed, as described in the 2018 European Society of Cardiology/European Society of Hypertension guidelines for the management of arterial hypertension) [25].
- Heart rate (HR) at rest.
- Cardiovascular medication record: therapeutic adherence was assessed using a personalized interview and by counting the number of pills between the inclusion visit and end of the program.
- Physical activity: pre- and postprogram physical activity levels were assessed using the International Physical Activity Questionnaire–Short Form (IPAQ-SF) [26].

#### Variables Related to Functional Capacity and Strength Assessment

The variables related functional capacity and strength assessment were as follows:

- METS at maximal effort and indirect anaerobic threshold
- HR at maximal exertion and indirect anaerobic threshold
- Systolic and diastolic blood pressures at maximal exertion and indirect anaerobic threshold
- The perceived sensation of exertion was assessed using the Borg scale at maximal effort and indirect anaerobic threshold [27].
- Lower- and upper-extremity strengths: sit-to-stand and arm curl tests were used to estimate maximal strength in 1 repetition with a linear transducer (encoder).

#### Variables Related to a Healthy Lifestyle

The variables related to a healthy lifestyle used in this work were as follows:

- Adherence to the Mediterranean diet: PREDIMED questionnaire
- Quality of life: EuroQoL questionnaire
- Anxiety and depression: HADS questionnaire
- Tobacco and other drug use
- Blood tests (measured in a fasting state as described in the European Society of Cardiology guidelines for the management of diabetes mellitus and dyslipidemia) [28,29]
- Glycosylated hemoglobin, total cholesterol, and low- and high-density lipoproteins

#### Variables Related to the Implementation of the Cardiac Telerehabilitation Program

The variables related to the program implementation were as follows:

- For the PT sessions, HR was measured using a Fitbit device range of devices with activity trackers, the Fitbit Ionic smartwatch, that transferred the data directly to the platform via Bluetooth with a 3- to 4-minute interval, and the sensation of perceived effort (Borg scale 1-10) [30] was recorded after questioning the patient at the end of each exercise series during the web-based training session.
- For the educational sessions, daily intake was recorded once weekly.

### Recruitment of the Patients: Inclusion and Exclusion Criteria

The study population included patients of both sexes, with no age limits, who had experienced an acute myocardial infarction in the previous 3 months; who had access to mobile phones, computers, or tablets with internet access; and who had not previously participated in a CRP (face-to-face or telematic). Conversely, patients who met the high-risk criteria for exercise-induced cardiovascular events according to the European guidelines for cardiac rehabilitation and exercise prescriptions for patients with cardiovascular diseases were excluded. Specifically, these criteria included left ventricular ejection fraction (LVEF) of <40%, New York Heart Association class >III, ischemia or significant arrhythmias documented during the inclusion stress test, and the placement of an implantable automatic device within 6 weeks before the start of the program. In addition, those with significant anemia (hemoglobin level <10 mg/100 mL), grade IV chronic obstructive pulmonary disease, or requiring home oxygen therapy were excluded from the study.

### Development and Implementation of the Program

#### Overview

During their first visit, the potential participants were evaluated for cardiovascular risk factors, adherence to the Mediterranean diet (PREDIMED questionnaire), and emotional status (HADS). In addition, quality of life and exercise training volume were evaluated using the EuroQoL and IPAQ-SF questionnaires, respectively.

Muscle strength was evaluated using the 30-second sit-to-stand (lower limb) and arm curl (upper limb) tests. The intensity prescribed was based on the basal characteristics of each patient and the maximum HR reached on the initial stress test on the treadmill using the Bruce protocol. In some cases with limited functional capacity, we used the modified Bruce protocol [31]. The 85% of maximum HR obtained in the stress test was the maximum HR allowed by the Fitbit device to guide the exercises, as described in the 2020 European Society of Cardiology Guidelines on Sports Cardiology and Exercise in Patients with Cardiovascular Disease [32].

**Figure 1:**
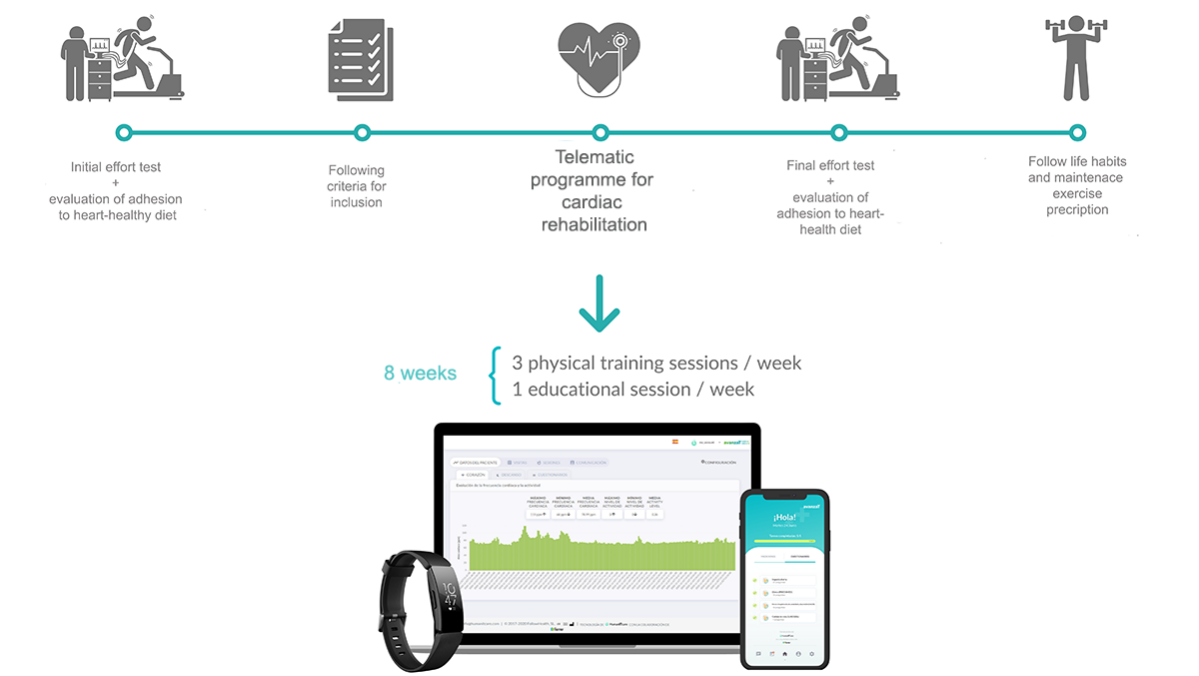
Scheme of the pilot cardiac telerehabilitation program.

The holistic CRP included 3 weekly exercise sessions and 1 weekly session regarding lifestyle habits, therapeutic adherence, and patient empowerment (Figure 1) for a duration of 8 weeks.

The sessions were performed by a physiotherapist or nurse expert in nutrition in groups of 8 patients. The sessions took place after working hours to increase patient adherence and to be compatible with normal hospital activities.

#### Exercise Sessions

Each participant completed 24 thrice weekly PT sessions, each lasting 1 hour. The sessions were conducted live via the platform, with guidance from a physiotherapist and cardiologist.

Exercise training consisted of aerobic and prescribed strength training based on the exercise testing and strength assessment; the HR and perceived exertion scale regulated the exercise intensity. The HR was monitored during the PT sessions using a Fitbit Inspire 2 device, which complied with the privacy shield policy. The training load was then increased overnight.

Patients directly contacted their assigned physiotherapist during and up to 30 minutes after the training sessions. In all sessions, there was a responsible cardiologist on call, in case of need. If any complication occurred within the indicated time, the physiotherapist initiated the medical emergency circuit, which consisted of contacting *112* (medical emergency telephone number) and detailing the complication to ensure prompt evaluation of the patient. The cardiologists were simultaneously informed of the adverse event.

**Figure 2:**
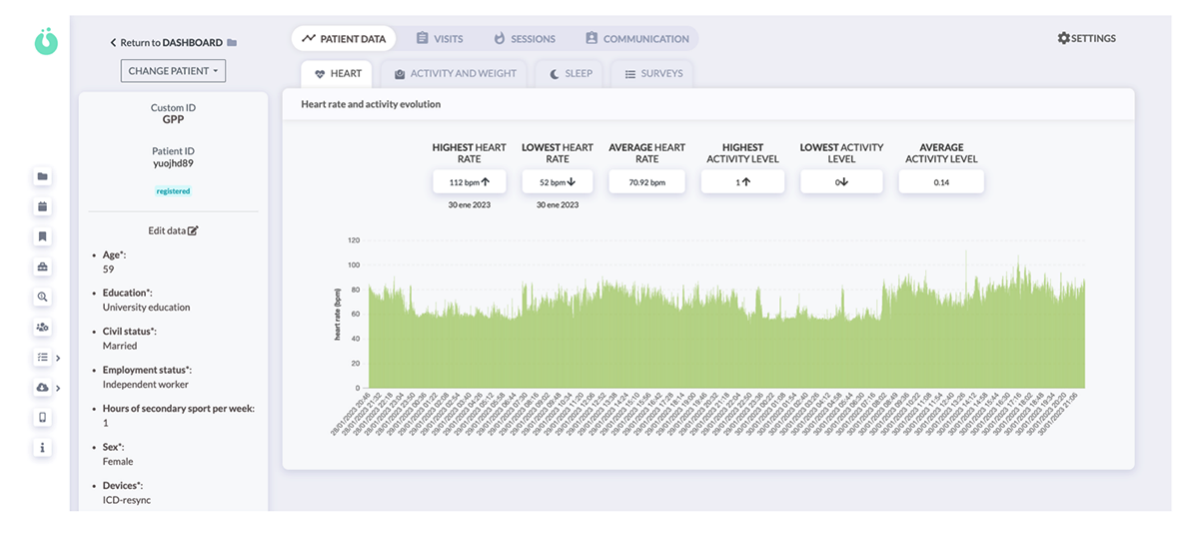
HumanITcare dashboard: heart rate over the time control panel.

All patients were instructed to bring along a family member during the PT sessions. In addition, before starting, all patients and their relatives (partners or caregivers) had to complete a workshop, which was offered live on the platform, on cardiopulmonary resuscitation and warning signs. Moreover, patients could forward their queries and messages through chat. The program physiotherapist reviewed these messages and questions daily and referred the patients to the medical team when necessary (Figure 2).

#### Educational Sessions

A specialized nurse accredited in nutrition conducted weekly group sessions through the web-based platform to address issues such as smoking, the Mediterranean diet, patient empowerment, therapeutic adherence, and psychological support, including strategies for acceptance of the disease, to comprehensively improve the quality of life and encourage a healthy lifestyle. In addition, all patients had individual monthly sessions during which the concepts covered during the group sessions were reinforced.

The last visit at the end of the program included a maximal exercise test: upper and lower extremity strength assessments. In addition, questionnaires assessing adherence to the Mediterranean diet, emotional state, quality of life, and medication count were also repeated.

#### Adherence to Exercise and Educational Sessions

We defined correct adherence if the patient completed the established and individualized protocol and the assistance was >80% of the session's educational and exercise.

### Statistical Analysis

Data were entered into an anonymized database, and statistical analyses were performed using Stata software (version 15.1; StataCorp LLC). Quantitative variables are expressed as mean (SD) or median according to their normality, as assessed using the Shapiro-Wilk test. Qualitative variables are expressed as n (%). Data were compared using Student 2-tailed *t* test for unpaired data if they followed a normal distribution and the Mann-Whitney *U* test if they did not. Statistical significance was set at *P*<.05.

### Subgroups Analysis: Sex and Age Differences

Our CRP included patients of both sexes with no age limit, so a comparison could be made in terms of feasibility; safety; and results in exercise, educational sessions, and adherence.

Designed Platform: HumanITcare Solution—Subjective Evaluation of the Patients of the CRP

The cardiac telerehabilitation program was carried out using the HumanITcare (SME Technology Co) platform [33], a telemedicine and telemonitoring platform for remotely monitoring chronic patients through integrated portable devices.

**Figure 3:**
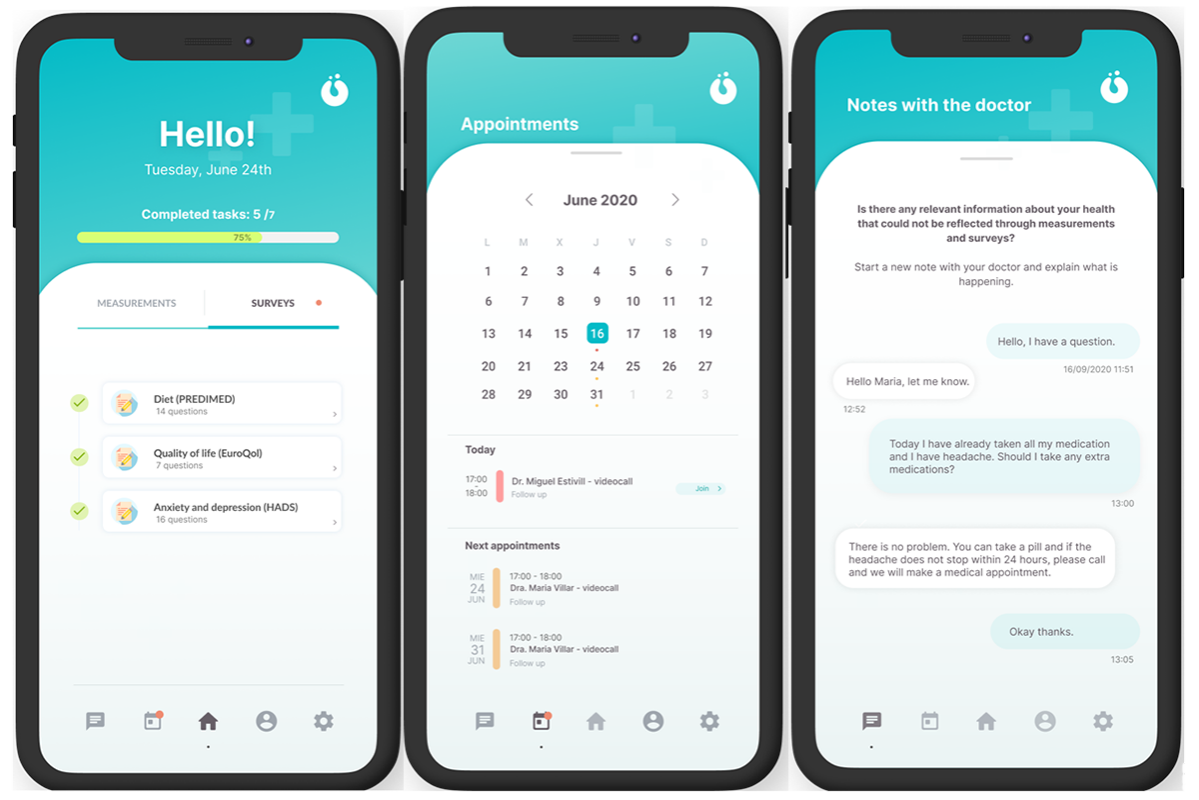
The app screen for the patient. PREDIMED: Prevención con Dieta Mediterránea.

HumanITcare is an application programing interface–based solution comprising a medical website for health care professionals and a mobile app for patients (Figure 3; available for Android and iOS). The website includes a medical portal to personalize each follow-up care plan, where medical professionals can access a dashboard with data collected from all patients. However, patients have to download an app to follow their care plans. The app allows automatic data collection through Bluetooth-linked sensors, integrated devices, and self-reported questionnaires. In addition, all data are transmitted to the cloud in real time; therefore, they can be analyzed, processed, and simultaneously displayed on the medical web portal.

Data security and privacy are essential to such solutions. Each patient had a username and a personal password to access the app. The company responsible for this platform is FOLLOWHEALTH S.L. (HumanITcare), which has servers in Ireland and complies with the General Data Protection Regulation of May 25, 2018, and the Data Protection Law (Organic Law on the Protection of Personal Data, Spain). Each participant in the study was assigned an ID consisting of 8 random characters (eg, d4w192bg), and participant data could only be reviewed using this ID.

Participant data were encrypted on the access platform for the researchers, technical administrators, and server. When the patient registered on the platform through the app, the following legal notice regarding data protection appeared on the screen to obtain consent from the patient for data processing:

This tool does not provide medical advice. It is only intended for information purposes for healthcare professionals. Do not use it as a substitute for professional medical advice, diagnosis, or treatment. Your doctor will review your responses and contact you, if necessary. Confidentiality of your data is important to us. Therefore, we have complied with the established data protection regulations. For more information, please read the detailed legal terms and conditions.

To improve satisfaction with the usability of the platform, feedback was collected from all users (patients and health professionals) during the study to enhance the different aspects of the tools used during the CRP, including reminders, bug reporting, and data visualization during sessions.

Material was provided to the patients to allow them to perform the program from home. This material included rehabilitation equipment and a wearable device to collect continuous data on patient habits during the program, specifically during the rehabilitation sessions.

After completion of the CRP, patients were also asked to complete a questionnaire to evaluate their satisfaction with the program, its duration, and the designed platform.

## Results

### Study Design, Variables Analyzed, and Recruitment of Patients

Between December 2020 and January 2022, 62 patients were included in the CRP; 2 were initially excluded (one because of ventricular arrhythmias during the initial stress test and the other because of technological incapacity). Therefore, 60 patients began the program, of whom 5 dropped out because of technical barriers and 5 dropped out because of schedule incompatibility.

Ultimately, 50 patients completed the program, 42 (85%) of whom were male, with a mean age of 58.9 (SD 10.3) years. In addition, the mean LVEF was 52.1% (SD 6.72%). According to the New York Heart Association functional classes, 50% (25/50), 40% (20/50), 10% (5/50), and 0% (0/50) of patients had classes I, II, III, and IV heart failure, respectively. No major or minor complications occurred during the course of the CRP (Figure 1).

### Development and Implementation of the Program: Exercise and Educational Sessions—Evaluation of Adherence

#### Exercise Sessions

CRP significantly improved the maximal and submaximal aerobic capacities, weekly training volume, and muscle strength. In addition, no changes in blood pressure values were observed at rest or during exercise (Table 1).

**Table 1:** Exercise stress test and muscle strength evaluation before and after the cardiac rehabilitation program (CRP) in the study population.

	Baseline participants, mean (SD)	Participants after the CRP, mean (SD)	*P* value
Maximal aerobic capacity (METs^a^)	8.3 (2.7)	9.8 (2.9)	.04
Weekly training volume (METs for week)	954 (629)	1757 (952	.03
METs VT1^b^	5.4 (1.6)	6.04 (1.7)	.03
METs VT2^c^	8.6 (2.3)	9.4 (2.5)	.04
Basal DBP^d^ (mm Hg)	76.99 (7.1)	75.7 (7.5)	.10^e^
DBP at maximal exercise (mm Hg)	78.4 (2.8)	78.4 (3.9)	.08
Basal SBP^f^ (mm Hg)	123.9 (15.49)	122.1 (11.4)	.09
SBP at maximal exercise (mm Hg)	168.1 (17)	167 (19)	.11
Arm curl test (repetitions)	19.4 (4.9)	22.4 (5.8)	.03
Sit-to-stand test (repetitions)	13.7 (2.8)	16.4 (3.9)	.04

^a^MET: metabolic equivalent task.

^b^VT1: first ventilator threshold.

^c^VT2: second ventilator threshold.

^d^DBP: diastolic blood pressure.

^e^Not significant.

^f^SBP: systolic blood pressure.

#### Educational Sessions

**Figure 4:**
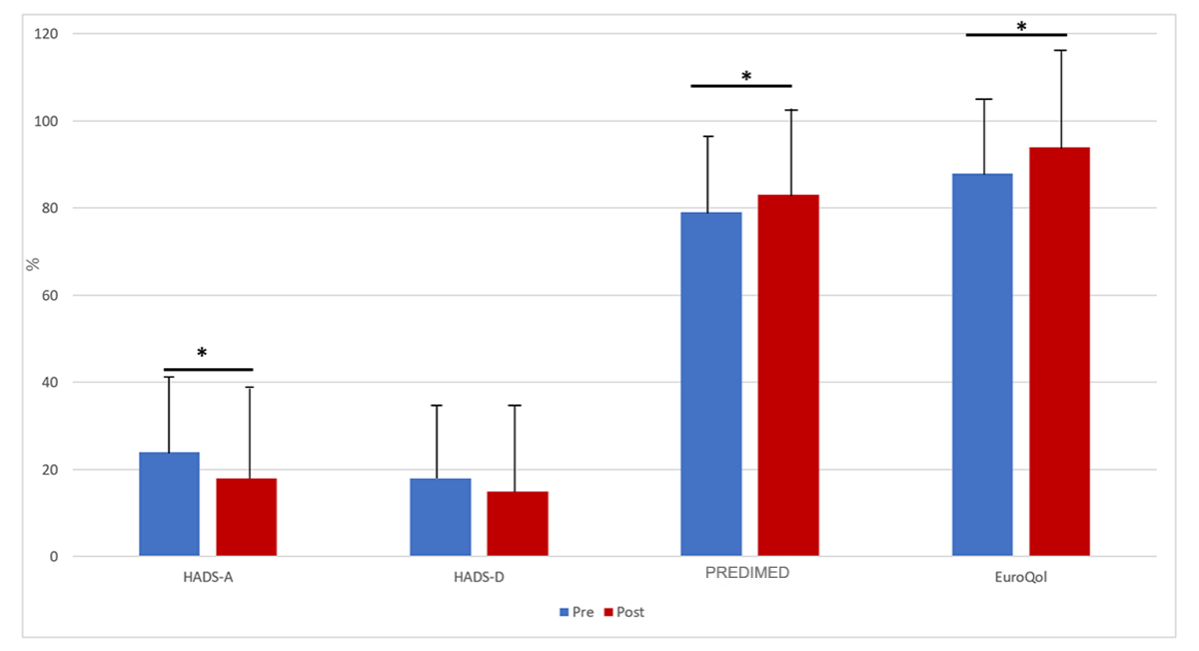
Adherence to the Mediterranean diet, emotional state, and quality of life before and after the program in the study population. *P*<.05 values significantly different between the indicated groups. HADS-A: *P*=.03; HADS-D: *P*=.09; PREDIMED: *P*=.04; EuroQol: *P*=.006. EuroQoL: European health questionnaire combining quantity and quality of life; HADS-A: Hospital Anxiety and Depression Scale–Anxiety; HADS-D: Hospital Anxiety and Depression Scale–Depression; PREDIMED: Prevención con Dieta Mediterránea—Mediterranean diet using the Mediterranean diet and lifestyle.

CRP significantly increased adherence to the Mediterranean diet, promoted a better quality of life, and improved the emotional state by reducing anxiety symptoms; however, no changes were observed in the depressive symptoms (Figure 4).

#### Adherence to Exercise and Educational Sessions

Adherence to the exercise (85.3%) and educational (86.6%) sessions was excellent. Additionally, there were no significant differences between male and female participants (exercise sessions: *P*=.10; educational sessions: *P*=.21; Table 2).

**Table 2:** Comparison between male and female participants in the adherence to nutritional and exercise sessions.

	Global participants, mean (SD)	Male participants, mean (SD)	Female participants, mean (SD	*P* value
Adherence to nutritional sessions (%)	86.6 (3.5)	84.2 (4.3)	96.7 (1.7)	.21
Adherence to exercise sessions (%)	85.3 (3.6)	82.9 (4.3)	95.6 (1.8)	.10

### Subgroup Analysis

#### Sex Differences

This CRP pilot study included 50 patients after an ischemic event, 42 (85%) of whom were male.

Subgroup analysis showed an equivalent impact of the CRP on maximal aerobic capacity and muscle strength. Similar results were observed for adherence to the Mediterranean diet, quality of life, and depression symptoms, with a greater impact on improving anxiety symptoms (Table 3).

**Table 3:** Comparison between male and female participants in the study population showed the increasing parameters of stress test, muscle strength, Mediterranean diet and lifestyle (Prevención con Dieta Mediterránea [PREDIMED]), Hospital Anxiety and Depression Scale (HADS), and EuroQoL evaluation before and after the cardiac rehabilitation program (CRP).

	Baseline male participants, mean (SD)	Male participants after the CRP, mean (SD)	Difference between before and after the CRP in male participants, mean (SD)	Baseline female participants, mean (SD)	Female participants after the CRP, mean (SD)	Difference between before and after the CRP in female participants, mean (SD)	*P* value of the difference between before and after the CRP in male vs female participants
Maximal aerobic capacity (METs^a^)	8.9 (0.4)	9.9 (0.6)	1.0 (0.2)^b^	6.9 (0.2)	7.8 (0.7)	0.9 (0.5)^b^	.14^c^
Weekly training volume (MET/wk)	1010 (112)	1900 (158)	890 (46)^b^	723 (93)	1169 (219)	446 (126)^b^	.21
METs VT1^d^	6.03 (0.5)	9.14 (0.6)	3.1 (0.2)^b^	4.2 (0.8)	5.9 (0.6)	1.7 (0.6)	.03
METs VT2^e^	8.9 (0.5)	9.6 (0.6)	0.7 (0.1)^b^	7.6 (1.3)	7.6 (3.1)	0.1 (0.0)	.04
Basal DBP^f^ (mm Hg)	76 (1.3)	75.1 (1.2)	−0.17 (0.2)	80 (0.9)	77.2 (0.7)	−3.2 (0.2)	.13
DBP at maximal exercise (mm Hg)	78 (1)	78.6 (0.8)	−0.6 (0.3)	80 (2)	77 (1.5)	−3 (0.5)	.31
Basal SBP^g^ (mm Hg)	121.8 (2.3)	121.2 (1.9)	−0.6 (0.4)	132 (6.8)	125 (3.1)	−7 (3.7)	.26
SBP at maximal exercise (mm Hg)	169 (3.1)	169 (3.0)	−0.2 (0.1)	162.2 (4.9)	158.9 (6.1)	−3.3 (1.2)	.28
Arm curl test (repetitions)	20.2 (0.8)	23.4 (0.9)	3.2 (0.1)^b^	16.7 (1.8)	18.7 (1.8)	2.0 (0.0)^b^	.11
Sit-to-stand test (repetitions)	14.1 (0.5)	17 (0.7)	2.0 (0.2)^b^	12.1 (1.1)	14.1 (1.7)	2.0 (0.6)^b^	.12
HADS-A^h^≥10 (%)	23 (2.4)	7 (1.3)	−15 (1.1)^b^	50 (2.1)	25 (1.70)	−25 (0.4)^b^	.03
HADS-D^i^≥10 (%)	12.9 (0.5)	7.8 (0.4)	−5.1 (0.1)	15 (8)	10 (1.9)	−5 (7.1)	.14
PREDIMED≥8 (%)	75 (0.5)	81.3 (0.8)	6.3 (0.6)^b^	100	100	0	.09
EuroQoL (%)	87.9 (2.8)	95.7 (2.9)	7.8 (0.1)^b^	82.2 (2.5)	89.2 (2.4)	7.0 (0.1)^b^	.08

^a^MET: metabolic equivalent task.

^b^Statistically significant result among the subgroups.

^c^Not significant.

^d^VT1: first ventilator threshold.

^e^VT2: second ventilator threshold.

^f^DBP: diastolic blood pressure.

^g^SBP: systolic blood pressure.

^h^HADS-A: Hospital Anxiety and Depression scale–Anxiety.

^i^HADS-D: Hospital Anxiety and Depression scale–Depression.

#### Age Differences

Among the 50 participants who completed the program, 6 (12%) were aged >75 years. In total, 45% (22/50) of the older population initially recruited dropped out because of technical issues, whereas none of the younger participants did so for this reason (Table 4).

**Table 4:** Comparison among participants aged <75 years and ≥75 years in the study population showed the increasing of different parameters.

	Baseline participants aged <75 years, mean (SD)	Participants aged <75 years after the CRP^a^, mean (SD)	Difference between before and after the CRP in participants aged <75 years, mean (SD)	Baseline participants aged ≥75 years, mean (SD)	Participants aged ≥75 years after the CRP, mean (SD)	Difference between before and after the CRP in participants aged ≥75 years, mean (SD)	*P* value of the difference between before and after the CRP participants aged <75 years vs ≥75 years
Maximal aerobic capacity (METs^b^)	8.9 (0.4)	9.9 (0.6)	1.0 (0.2)^c^	6.9 (0.2)	7.8 (0.7)	0.9 (0.5)^c^	.14^d^
Weekly training volume (METs per week)	1010 (112)	1900 (158)	890 (46)^c^	723 (93)	1169 (219)	446 (126)^c^	.21
METs VT1^e^	6.03 (0.5)	9.14 (0.6)	3.1 (0.2)^c^	4.2 (0.8)	5.9 (0.6)	1.7 (0.6)	.03
METs VT2^f^	8.9 (0.5)	9.6 (0.6)	0.7 (0.1)^c^	7.6 (1.3)	7.6 (3.1)	0.1 (0.0)	.04
Basal DBP^g^ (mmHg)	76 (1.3)	75.1 (1.2)	−0.17 (0.2)	80 (0.9)	77.2 (0.7)	−3.2 (0.2)	.13
DBP at maximal exercise (mmHg)	78 (1)	78.6 (0.8)	−0.6 (0.3)	80 (2)	77 (1.5)	−3 (0.5)	.31
Basal SBP (mmHg)	121.8 (2.3)	121.2 (1.9)	−0.6 (0.4)	132 (6.8)	125 (3.1)	−7 (3.7)	.26
SBP^h^ at maximal exercise (mmHg)	169 (3.1)	169 (3.0)	−0.2 (0.1)	162.2 (4.9)	158.9 (6.1)	−3.3 (1.2)	.28

^a^CRP: cardiac rehabilitation program.

^b^MET: metabolic equivalent task.

^c^Statistically significant result among the subgroups.

^d^Not significant.

^e^VT1: first ventilator threshold.

^f^VT2: second ventilator threshold.

^g^DBP: diastolic blood pressure.

^h^SBP: systolic blood pressure.

### Designed Platform: HumanITcare Solution (Subjective Evaluation of the Patients of the CRP, Its Duration, and the Designed Platform)

Upon completion of the CRP, 78% (39/50) of the study population reported improved functional capacity and therapeutic adherence. In addition, 56% (28/50) of the participants stated that they were much closer to a healthier lifestyle and found themselves less anxious and more empowered about their disease.

Moreover, all patients reported a good rapport with the CRP team and considered the management of the program to be excellent.

Regarding CRP duration, 63% (31/50) of the patients considered it adequate, whereas the remaining 37% (18/50) preferred a longer program. In addition, 29% (14/50), 34% (17/50), and 37% (18/50) of patients showed a preference for face-to-face, telematic, and hybrid strategies, respectively.

Regarding the usability of the designed platform, 51% (26/50) of the patients described the platform as easy to use, whereas 34% (17/50) and 15% (7/50) faced minor and significant technical problems, respectively. In addition, 83% (41/50) of patients aged >75 years and 40% (20/50) of younger patients faced technological difficulties when using the platform.

## Discussion

### Principal Findings

#### Overview

In this pilot study, we developed a holistic telematic CRP dedicated to patients after an ischemic cardiac event, including patients of both sexes with no age limit. Our results can be summarized into four key findings: (1) telematic CRP, including tailored aerobic and strength training sessions, improved maximal and submaximal aerobic capacities and muscle strength; (2) a holistic approach, including educational sessions and emotional support, significantly improved adherence to the Mediterranean diet, emotional state, and quality of life; (3) CRP was beneficial irrespective of age and sex; and (4) the telematic CRP strategy was safe and feasible for our study population, although there were differences in the preferences of patients in terms of duration and type of strategy.

#### Exercise Sessions: Improvement in Functional Capacity and Muscle Strength

In the overall study population, our telematic CRP led to an improvement of 1.6 METs. This increase is equivalent to the reported face-to-face CRP conducted in comparable study populations and with a similar training protocol (a combination of aerobic and resistance training) [34]. In contrast, it was superior to that observed after CRPs that involved only independent aerobic PT during the telematic or face-to-face strategy. Strength training is safe for patients with ischemic heart disease and is synergistic with aerobic exercise training [35]. Furthermore, our PT protocol significantly increased the results of the arm curl (15.5%) and sit-to-stand (19.7%) tests, indirectly measuring the arm and leg muscle strength resistance, respectively. Both aerobic and strength training are essential for exercise-induced beneficial metabolic effects [36]. In addition, muscle strength is known to be strongly correlated with quality of life and autonomy, especially in older adult patients [37]. Therefore, on-site or telematic strength training should be part of all PT protocols in CRPs.

#### Educational Sessions: Adherence to Mediterranean Diet, Emotional State, and Quality of Life

Our comprehensive telematic CRP included nutritional counseling, educational sessions, and emotional support. These interventions enhance patient empowerment by promoting a healthy lifestyle [38,39]. In a recent meta-analysis [40], CRP reduced anxiety and depression rates without differences between face-to-face and telematic strategies. In our study, patients were made cognizant of their disease, after which their anxiety levels significantly decreased. However, no significant changes were observed in depressive symptoms. A meta-analysis reported that the prevalence of depression after acute coronary syndrome in round 14 improved to 3% after cardiac rehabilitation [41]. However, the COVID-19 pandemic increased this percentage to 34% [42]. Our study population had average depression rates of 16.9% preintervention and 15% postintervention; therefore, we hypothesize that this nonsignificant improvement in the post-CRP depression score could have resulted from the COVID-19 pandemic. Meanwhile, anxiety levels positively and significantly improved after the program (23% preintervention and 17% afterward), possibly because of greater empowerment of the patient and improved cognition regarding their disease.

In compliance with the previously mentioned meta-analysis, our holistic CRP, including individual and group nutritional counseling, significantly improved compliance with the Mediterranean diet, which is the best-studied and most evidence-based diet for preventing ischemic events and is considered the gold standard for healthy eating [43]. Moreover, it reduces the risk of repeated cardiovascular events, as evidenced by a previous randomized controlled trial [44]. Therefore, CRP must include nutritional assessments, possibly with the PREDIMED score, and more complements to medical treatment.

Furthermore, home-based CRP significantly improved quality of life, concordant with similar studies on telematic cardiac rehabilitation [45] and center-based CRP [46]. This may be because of patient empowerment regarding their cardiac condition, which allows them to resume their previous daily activities without fear and provides tools for the management of cardiac symptoms and stress.

#### Sex Differences: Feasibility and Safety of the Telematic CRP

This CRP pilot study included 50 patients after an ischemic event, 42 (85%) of whom were male. This percentage was similar to that of other studies, and US surveys have shown that following a heart attack, 14.3% of female individuals participated in CRPs compared with 22.1% of male individuals [47]. Female individuals are less likely to engage in CRP than male individuals because of less encouragement from practitioners and psychosocial barriers, such as familial obligations, lack of support, and misperception of the disease [48]. Furthermore, they are usually older and have more comorbidities, increased anxiety and depression, worse functional capacity, and worse CRP results, compared with male individuals [15]. Specific interventions, such as automatic referral, strong physician recommendation, psychological support, and home-based or tailored programs, can increase adherence and improve results in female individuals [49,50].

Our pilot telematic CRP attempted to provide the flexibility of a tailored intervention for female patients and psychological support, which resulted in an equivalent impact of CRP on maximal aerobic capacity and muscle strength and better impact on improving anxiety symptoms (Table 2). Although female patients had higher levels of anxiety preintervention, it could be easier in this group to achieve positive scores, highlighting the special importance of psychological sessions in female patients. Conversely, CRP adherence was similar among male and female patients (Table 3), which differed from the superior adherence observed among male individuals in traditional center-based studies [51].

#### Age Differences: Feasibility and Safety of the Telematic CRP

Of the study participants, 12% (6/50) were aged >75 years and 24% (12/50) had an LVEF of <50%. Although both represented a small percentage of the entire group, these patients had no complications during the intervention. However, CRP use among older patients remains low despite evidence suggesting lower mortality, hospitalization rates, and Medicare costs and improved symptomatology [52]. This may be because of multiple comorbidities; psychosocial factors, such as denial of disease severity and depression; and other difficulties, such as transportation to the CRP centers [53]. Newer CRP models include home-based approaches to overcome these barriers [54]. In addition, CRP benefits patients with low LVEF [55], in whom exercise training is not associated with adverse effects on left ventricular remodeling, and most patients have improved functional capacities despite the LVEF [56]. Both older patients and those with a low LVEF and poor functional class are associated with frailty, which is increased in patients who undergo CRP for acute coronary syndrome [57].

In the subgroup analysis (Table 4), patients aged >75 years had an increase of 1.7 (SD 0.9 METs) in maximal aerobic capacity, which reached statistical significance despite the small study subgroup. Furthermore, CRP induced significant improvements in adherence to the Mediterranean diet and emotional state, and these improvements were better than those observed in younger individuals. No major or minor complications occurred during the intervention in this subgroup. Moreover, no minor or major complications were observed in this study population. Although these results are promising in terms of positive values of CRP for the older population, the feasibility of the telematic approach is low, as 45% of the older population recruited for the program dropped out because of technical issues, whereas none of the younger participants did so for this reason. Overall, these results emphasize that CRP for older patients must be tailored to individual clinical complexities to obtain better outcomes of functional capacity, nutritional status, comorbidities, cognitive status, and socioeconomic support.

#### Designed Platform: HumanITcare Solution (Subjective Evaluation of the Patients of the CRP, Its Duration, and the Designed Platform)

Regarding the designed platform, 50% (25/50) of the patients adequately handled it; however, 15% (7/50) still required help. In addition, 83% (41/50) of patients aged >75 years and 40% (20/50) of younger patients faced technological difficulties when using the platform.

Feedback collected from patients and health professionals during the study could enhance the different aspects of the tools that have already been implemented, whereas others will soon be incorporated. In this manner, our solution can be better adapted for people with different technological backgrounds, allowing the inclusion of more patients in such telematic programs.

However, wearable devices do not provide real-time data, which are crucial for monitoring the maximum HR of a patient during telematic rehabilitation sessions.

Therefore, we plan to incorporate new wearable devices into our platform in the future to resolve this problem. If different brands of wearable devices are available to collect data on the platform, patients may already be using them; thus, offering a “bring your own device” approach would be helpful. In addition, some of these devices allow third-party apps to record HRs in real time or with minor delays.

Regarding the preferences for CRP strategies, one-third of the patients preferred the face-to-face approach, one-third preferred the telematic approach, and one-third were comfortable with both strategies. These results emphasize the need for a tailored CRP program that offers different approaches and durations based on patient characteristics and preferences.

Finally, regarding the duration of CRP, one-third of our study population considered a longer CRP necessary for better results. Phase 3 (maintenance) intervention, usually developed between general practitioners and the cardiology team, helps patients maintain their physical activity levels and reduces the risk of new cardiovascular events [58]. However, costs, coordination among different teams, and patient motivation are crucial for its development, which remains a challenge for further improvement.

### Comparison With Prior Work

As mentioned above, there are few studies in the literature about the telematic cardiac rehabilitation approach, which is effective and safe for patients following an ischemic event [16]. However, most of them came from selected populations, excluding female patients [15], patients aged >75 years [17], and patients with multiple comorbidities [18].

In this study, we developed a cardiac telerehabilitation platform, specifically designed for patients after a cardiac ischemic event, with no sex or age limitation. The CRP included PT (aerobic and strength) and interventions for improving the emotional sphere and adherence to a healthy lifestyle.

### Strengths and Limitations

This pilot CRP after an ischemic cardiac event configures a holistic approach. It includes patients of both sexes with no age limit and is safe in all subgroups. It achieved positive results in improving maximal aerobic capacity, weekly training volume, muscle strength, quality of life, compliance with diet, and anxiety symptoms.

This study has a few limitations. First, the small sample size of the patients included in this pilot study limited statistical power. Second, only 15% (7/50) of the participants were female and 12% (6/50) were older patients; therefore, our results regarding the impact of age and sex on the beneficial effects of CRP should be considered cautiously. Regarding these 2 facts, this was a small study but included patients of all ages and both sexes, being safe and achieving positive results in all subgroups. It is necessary to corroborate these results in larger programs.

Third, follow-up was not conducted; thus, the evolution of functional capacity, adherence to a healthy lifestyle, and clinical events following the completion of the program remain unknown. Nevertheless, these data combination allows a better understanding of the medical conditions of the patients, thereby improving the diagnosis or treatment provided. An algorithm that enables patients to make lifestyle recommendations, thereby improving their quality of life after acute myocardial infarction, can be implemented.

Finally, when it came to including patients in the program, a significant limitation was at the technological level. Patients needed a cell phone with an updated operating system to access all functions of the app accurately. In addition, patients required basic skills to manage the platform or an environment that could support them in this regard. Although the technological education of the older population is improving, there is still a gap with that of the current generation.

### Conclusions and Future Directions

Moreover, most of them do not have a holistic approach as they do not incorporate interventions with demonstrated positive impact on CRP, such as the emotional sphere [19], nutrition [20], and medication adherence [8]. Moreover, exercise sessions do not include strength training, with synergic effect with aerobic exercised.

Future studies with more patients will be needed, with a higher percentage of individuals who are fragile and aged >75 years, as well as female patients. Improvements to the technological approach should be made. The preference for a face-to-face or telematic CRP varied among the study population, which emphasizes the need for a tailored CRP offering telematic, on-site, and hybrid models to improve adherence and completion rates. Short- and long-term follow-up should be performed to confirm these results.

## Abbreviations

CRP: cardiac rehabilitation program
HADS: Hospital Anxiety and Depression Scale
HR: heart rate
IPAQ-SF: International Physical Activity Questionnaire–Short Form
LVEF: left ventricular ejection fraction
MET: metabolic equivalent task
PREDIMED: Prevención con Dieta Mediterránea
PT: physical training

## Data Availability

The data sets used and/or analyzed during this study are available from the corresponding author upon reasonable request.
